# The Hospital Sunday Fund

**Published:** 1912-03-16

**Authors:** 


### THE HOSPITAL SUNDAY FUND.

### THE LORD MAYOR'S REPORT ON THE ADMINISTRATION OF ST. GEORGE'S HOSPITAL.

A Special Meeting of the Council of this Fund was held at the Mansion House on Thursday last, March 7, under the chairmanship (owing to the temporary indisposition of the Lord Mayor) of Sir John Bell, Bart., in fulfilment of an undertaking made to the annual meeting of the Fund by the Lord Mayor to inquire personally into the questions which had been raised as to the administration of St. George's Hospital.

After the formal business of reading the notice convening the meeting, the reading of letters of apology for absence, and the passing of the minutes, the Secretary, Sir Edmund Currie, read the Lord Mayor's report of his investigation, as follows :—

### The Lord Mayor's Report.

The Committee of the King's Hospital Fund have withheld their grant to St. George's Hospital on the ground that the amount expended upon the bacteriological and pathological departments is excessive, and much larger than the proportion spent thereon at other hospitals.

In order to carry out my undertaking at the last meeting of the Metropolitan Hospital Sunday Fund, I visited the hospital on February 12, when I was met by the treasurer and some of the house committee, who placed before me an account of the work done in these departments, the cost of their maintenance, and the amount represented by that cost per occupied bed.

Statistics obtained from other hospitals showing this proportion were also produced for comparison. It was pointed out that the work of these departments is essential for the proper diagnosis and treatment of the patients of the hospital; that it is necessarily undertaken by skilled experts, and that in St. George's Hospital all reports on clinical material are furnished personally by the bacteriologist and pathologist to the hospital, and not by students or even by house physicians or house surgeons.

The work includes examinations of blood, excretions, sputum, etc., post-mortem examinations, and the examination of various growths and tissues, the preparation of vaccines, etc., besides the care of the museum, which is the property of the hospital, also a variety of chemical examinations.

A careful investigation of the whole matter convinced me that the bacteriological and pathological departments of St. George's Hospital are doing work which is recognised at all efficient hospitals as essential to the scientific diagnosis and treatment of disease; that the amount expended upon it is in no way excessive and does not represent a larger cost per occupied bed than is the case at many other London hospitals, although as the accounts of these departments are not kept in a corresponding manner at different hospitals (various items being excluded or included) it is difficult to show this in exact figures.

The work of these departments is as necessary to the welfare of the patients as that of the electrical department, the *x*-ray investigations, or the preparation of drugs, and it involves most laborious processes which can only be efficiently carried out by skilled and highly-trained officers with appropriate salaries.

I am of opinion, after an investigation of all the facts, that the proportionate amount paid by the hospital and school for the upkeep of the laboratories is a fair and proper one, and that the sums expended upon these departments by the hospital are properly taken from the general funds, being for the direct treatment of the patients.

(Signed) Thomas Boor Crosby, Lord Mayor.

The Chairman, in asking whether any member of the Council wished to comment upon the report, said he thought it would be agreed that very hearty thanks should be tendered to the Lord Mayor for what he had done. It was a matter of regret that indisposition prevented his attendance.

### The Discussion.

Mr. West, Chairman of St. George's Hospital, said that the hospital welcomed the visit of the Lord Mayor to St. George's, when he went into matters very fully and carefully, and they were very grateful to him for the report which he had furnished.

Rev. Canon Pearce asked what was the next procedure to follow in regard to the report.

The Secretary said it occurred to him that it would be a great pity if the two Funds were at variance on the matter. It was well known that in this matter King Edward's Fund differed from the Hospital Sunday Fund, as the latter had made its grant to St. George's Hospital, while the King Edward's Fund required the medical school of St. George's to repay to the hospital a certain sum of money. The Lord Mayor paid his visit to St. George's, as promised to the constituents of the Fund at the last meeting, without anyone coaching him; and the report showed that what St. George's Hospital had done was correct, and he (the Secretary) suggested that perhaps there was some way in which the money could be paid out of the St. George's discretionary fund, so as to relieve the difficulty. At some of these large hospitals there was a discretionary fund, under which subscribers sent their money to the committee, and left it to them to spend it on either the hospital or the medical school as they wished. St. George's Hospital had plenty of money in its discretionary fund to enable them to pay out of it the amount in question. He wondered whether St. George's Hospital Committee could carry out this suggestion. That would probably prevent there being any difference of opinion between the two Funds.

Rev. Canon Pearce said the suggestion of the Secretary assumed that the question was settled, but he was not sure of that. His feeling was that the Council should not be satisfied with something which did not satisfy the King's Fund. He would like this correspondence and report to go to the King's Fund as a matter of courtesy.

The Secretary said that during the many years of his connection with the Fund there had never been any difference of opinion between it and the King's Fund. And he felt that if both Funds were working for the same object they should pull together. Unlike the King's Fund, this Fund did not go into details of management before making the grant, and a grant was not withheld unless there was a scandal or definite trouble.

Mr. Norman considered that the Lord Mayor's report had justified the course which the Council had taken, and he did not see that it was for this Fund to move in the matter. Possibly as a result of this report the King's Fund might in future alter its practice in this respect. Perhaps an intimation might be made that the Council of the Sunday Fund proposed to do the same as in the past.

Mr. Parker Young thought it would be desirable, if possible, to get from Mr. West a statement of the proportionate amount paid to the hospital and to the medical school. The separate amounts were not stated in the report, and if they were given it could be seen whether they corresponded to those at other hospitals.

Mr. West said St. George's Hospital Committee would gladly welcome any means by which it could fall in with the views of the King's Fund in the matter. A meeting of the King's Fund took place, when it was publicly stated, and got into the Press, that they were not satisfied, and that St. George's Hospital was devoting money, given to the general fund for the sick poor, to the support of medical education. That compelled St. George's Hospital to take means to justify what it had done. If this Council would give him its views in the form of a resolution, he did not doubt that his committee would do accordingly.

The Chairman pointed out that such a resolution would probably end the unpleasant incident and the equally unpleasant meetings which arose out of it.

Sir Dyce Duckworth considered it very important that this Council should make it plain to the public that the expenditure of the money in question, wherever it came from, was entirely for the benefit of the patients of the hospital. There was in some quarters a gross and popular idea that the money was given to medical students, or other people, for part of their education. But as a matter of fact the gifts were intended to help forward the exact knowledge of disease which the patients were suffering from, which exact knowledge was an essential requirement of modern times. It was very opportune to insist that the money was well spent, and was for the good of individual patients.

Mr. Norman having stated his willingness to submit a resolution,

Sir William Church expressed his pleasure that a resolution would be submitted, as it was likely to assist in clearing matters. But there was a little difficulty, as some hospitals did not possess a discretionary fund. He had had to give a great deal of time to this matter, and it was difficult to apportion the expenses to the hospital and to the school, and he did not know upon what ground the Lord Mayor reported that the cost at St. George's did not represent a larger amount per occupied bed than at many other London hospitals.

Rev. Dr. Fleming pointed out that the desire was to make peace with the constituents of the Fund rather than with the King's Fund, and he was not sure whether the constituents would be prepared to accept the award of the Lord Mayor. If the meeting of constituents acquiesced in the investigation, that was an end of the matter, as far as the Council was concerned.

The Chairman was not prepared to say the constituents would accept the Lord Mayor's report, or any report; but they gladly agreed to the Lord Mayor's promise of personal attention.

Mr. Norman pointed out that the desire was not to make an agreement with the King's Fund, but to suggest an arrangement by which the criticism of the King's Fund would be removed, and which would be agreeable to the King's Fund.

Sir A. Pearce Gould remarked that the question was twofold. Some objected to the expenditure on St. George's Hospital because it seemed excessive, and that was the objection of the King's Fund. But the subscribers to the Hospital Sunday Fund objected not because it was excessive, but because the money had been taken from the general fund. Those matters should be kept separate. The resolution which it was intended to move would be likely to meet the grievance of the subscribers, as the amount which St. George's Hospital thought fit to pay out of the discretionary fund was not a matter with which the subscribers to this Fund could be deeply concerned. Personally, he felt grateful to St. George's Hospital for having made an effort to remunerate its expert advisers somewhat appropriately.

Mr. Norman moved: "That in consideration of the Lord Mayor's report with respect to St. George's Hospital, with the view to securing, as far as possible, uniformity of practice between King Edward's Fund and the Hospital Sunday Fund, the committee of St. George's Hospital be invited to defray, if possible, the expenses of the bacteriological and pathological departments out of their discretionary fund.

Sir William Church said he had no right to speak for the King's Fund, or to represent the views of those gentlemen. The trouble arose from St. George's Hospital, without notice or warning, ceasing to enter in the unified system of accounts their discretionary fund at all, and paying it all to the general fund. In their own handbook there was a note stating the amount which was paid to the discretionary fund, and that amount was very much larger than the amount which was paid by the hospital to the school for work done. Therefore the distribution committee of the Sunday Fund was satisfied they worked upon the note in the handbook. The public had never made any objection to the payments by St. George's to the bacteriological and pathological departments so long as they also saw that they had a discretionary fund which was much larger in amount than the payments made. He did not see any reason for the Hospital Sunday Fund Council sending up such a resolution to the King's Fund.

Rev. E. S. Hilliard said that as Mr. West had heard all that had been said, and would probably be prepared to lay before his committee what had transpired, he would no doubt press the view of the Council on that committee. Therefore, perhaps the best course would be to accept with thanks the report of the Lord Mayor, who had thus carried out his promise to the constituents, and leave Mr. West to do what was right in the light of that discussion.

Mr. Norman withdrew his resolution, with consent, and the Rev. Mr. Hilliard's suggestion was adopted.

### St. John's Hospital for Skin Diseases.

The Secretary reminded the Council that at the last meeting he stated a letter had been received from a late officer of this hospital preferring serious charges against the hospital. The letter was referred to the General Purposes Committee. That committee invited the attendance of two members of the hospital, with the secretary, to a discussion on the matter. The secretary of the hospital replied referring the committee to their solicitors. The solicitors wrote to ask what charges were made. He (the Secretary) replied to that, stating that what was wished was a friendly talk. Subsequently he received a letter from the solicitors saying that a writ had been served against the hospital. He (the Secretary) therefore wrote and ended the matter, as he felt it was not the desire of the Council to be concerned with legal complications.

### The Death of Mr. John S. Gilliat.

The Chairman referred with regretful feelings to the death of Mr. John Gilliat, and proposed that a resolution of condolence be forwarded to his family. The deceased had been a great supporter of this and many other institutions.

